# High intensity focused ultrasound *vs.* cryotherapy as primary treatment for prostate cancer

**DOI:** 10.4103/0970-1591.38597

**Published:** 2008

**Authors:** Pratyush Ranjan, Gyan Saurabh, Rahul Bansal, Amit Gupta

**Affiliations:** Department of Surgical Disciplines, All India Institute of Medical Sciences, New Delhi, India

**Keywords:** Cryotherapy, high intensity focused ultrasound, prostate cancer

## Abstract

Prostate cancer is one of the most commonly diagnosed cancers. Here, we will be discussing two upcoming techniques for its management. One is cryotherapy which has returned from oblivion after nearly 150 years armed with latest technology and looking as if its full potential has been recognized now. On the other hand is high intensity focused ultrasound (HIFU), the application of ultrasound to this field is relatively new and hence a lot of excitement and hope.

We searched MEDLINE (PubMed 1942-2005), reference lists of retrieved articles, urology textbooks and our own data looking for studies comparing cryotherapy and HIFU. From 81 titles or abstracts, two independent reviewers identified 50 as potentially relevant. Disagreement was resolved by discussion involving the third reviewer and we finally identified 45 articles. Full reports of 45 articles were retrieved and final selection was made by the same two independent reviewers using the same criteria as for the initial selection. Data were extracted and methodological qualities of selected studies were reviewed by two independent reviewers. Qualitative analysis and synthesis were done.

Treatment options depend upon the age of patient, grade of tumor and expectations out of treatment. Patient choice governs the treatment actually to be given. It is the selection of a patient for a particular treatment option that decides how favorable the outcome is going to be. Both these techniques are relatively new and they look promising but both lack long-term data to prove their efficacy.

## INTRODUCTION

Prostate cancer is one of the most commonly diagnosed cancers and with the number of people being diagnosed as having prostate cancer on the rise, the economic burden is going to be immense. There is a need to find a potential treatment which cuts on cost without compromising on quality and to put our resources to develop it further.

Treatment options for the clinically localized prostate cancer include radical prostatectomy, radiation therapy (both external beam radiation therapy [EBRT] and/or brachytherapy) or watchful waiting. Minimally invasive techniques have generated a lot of enthusiasm and interest as they involve a shorter hospital stay. Despite a galaxy of these techniques, the quest to find the perfect one is still on. We will be discussing two techniques which have generated a lot of excitement among practicing surgeons. One is cryotherapy which has returned from oblivion after nearly 150 years armed with latest technology and looking as if its full potential has been recognized now. On the other hand is HIFU; the application of ultrasound to this field is relatively new and hence a lot of excitement and hope.

We first performed a MEDLINE search (Pubmed 1942 to 2005) using the terms “Cryotherapy”, “HIFU” and “Carcinoma Prostate”. Two independent members (PR and GS) of the research team reviewed all titles and available extracts identified after the MEDLINE search. Out of 81 articles retrieved, the majority were in English except four in Spanish (two of cryotherapy and two of HIFU), six in German (five of cryo and one of HIFU) and one in Czech (of cryo), three in French (one of cryo and two of HIFU), one in Italian (of cryo) and one in Japanese (of HIFU). We could use only Spanish and Japanese articles [courtesy one of our member (RB)] in our study, the rest were excluded. Finally, reviewers agreed on potential relevance only in 50 articles. Disagreement was resolved by discussion involving a third reviewer (AG) and we finally selected 45 articles, identified through MEDLINE (n = 42), reference of retrieved articles (n = 2) and urology textbook (n = 1). Data was extracted and methodological qualities of selected studies were reviewed by two independent reviewers (PR and GS).Qualitative analysis and synthesis was done.

## CRYOTHERAPY

This technique was introduced long back; the earliest reported application is from 19^th^ century London where Arnott applied ice salt mixture to the cancer of the breast and cervix.[1] In 1966 the advent of probes cooled by liquid nitrogen in closed circulation marked the beginning of modern cryotherapy[2] and one of the first application of this new technology was the transurethral cryoablation of benign prostatic hyperplastic tissue,[3] soon followed the treatment of prostate cancer via an open perineal approach.[4] The transperineal approach was introduced in 1974, initially using a single digitally guided cryoprobe repositioned as needed during the procedure.[5] As the procedure was associated with significant side-effects, it was abandoned until early 1980s. It was reintroduced by Onik and Cohen in 1988 taking advantage of several technical advances of that time.[6]

The introduction of real time Transrectal Ultra Sound [TRUS] guided placement of the cryoprobes and continuous monitoring of the ice ball progression resulted in revival of interest in cryosurgery. The reintroduction of several significant technical and procedural advances have occurred including the development of vacuum-insulated cryoprobes, use of argon gas instead of liquid nitrogen in the probes, the evolution of intraoperative treatment planning systems, the introduction of systemic temperature monitoring and most recently the development of a temperature feedback automated freezing system,[7] making cryosurgery a potential technique for the future.

Historically, cryotherapy treatment was indicated mainly as a salvage procedure for local recurrence following radiotherapy but more recently it has been used as a primary treatment for patients with localized or locally advanced prostate cancer.

Cryoablation is best performed for glands smaller than 40 g; therefore some authors[8] recommend neoadjuvant androgen deprivation (three to eight months) to downsize the prostate.

### Indications for Cryoablation[9–12]

1. Localized prostate cancer in high-risk patients with contraindications for radical prostatectomy, including patients refusing other forms of therapy.
2. Salvage cryotherapy for patients in whom radiation therapy (either external radiation or brachytherapy) has failed.
3. Local recurrences after radical prostatectomy.
4. Prostate volume of < 40 mL.
5. Debulking of large primary tumors with or without metastatic disease.
6. High-stage tumors (if the initial stage of tumor is cT2b or more, the chances of margin involvement is significant, cryoablation is effective because of its ability to freeze tissue outside the surgical margins).
7. High-grade tumors (local treatment with cryoablation can be achieved with systemic treatment being reserved for those who demonstrate need).
8. Patients who have undergone prior radiation for rectal carcinoma.
9. Patients who are Jehovah witness.
10. Patients who are non-surgical candidates with significant lower urinary tract symptoms.
11. Patients who need persistent anticoagulation.

### Contraindications

Cryoablation may be contraindicated in patients with

1. Advanced local disease.
2. Incontinence.
3. History of anorectal fistula formation secondary to inflammatory bowel disease.
4. Prior transurethral resection of prostate (the sloughing rate in these patients is higher than non-resected patients).

Additionally it is recommended that patients with a PSA level greater than 15 ng/ml should undergo pelvic lymph node dissection before undergoing definitive cryosurgical procedure. Furthermore the use of androgen blocking agents increases the deposition of fat in Denovilliers' fascia making freezing less likely during the procedure.[13]

### Procedure

Cryotherapy is performed under general or regional anesthesia. The current third generation cryoablation system uses argon gas for freezing and helium for rewarming, temperature monitoring within and outside the prostate and a standardized urethral warming catheter. The use of gaseous element has allowed substantial downsizing of cryoprobes (17 gauge, 1.5 mm), making more rapid freeze-thaw cycle possible. The smaller cryoprobes allow placement of a larger number of probes through a 17 gauge interstitial radiotherapy template.

After anesthesia the patient is placed in dorsal lithotomy position and prostate is mapped with TRUS. Four to eight cryoprobes are introduced through the perineum and advanced to preselected locations in the prostate gland using ultrasound guidance. Temperature monitoring probes are also placed percutaneously through the perineum. Depending upon the preference and experience of the surgeon, up to five thermocouples may be placed in the mid-gland, at the level of the external sphincter, Denovilliers' fascia, the left and right neurovascular bundle. Thermosensors at the level of the external sphincter and Denovilliers' fascia are used to minimize the risk of incontinence or recto-urethral fistula, while those in mid-gland and the neurovascular bundles ensure that the required temperature of -40°C is reached. Flexible cystoscopy is done to ensure that none of the needles has inadvertently pierced the urethra. A warming catheter is then inserted into the urethra to prevent it from being damaged by cold with continuous flow of warm fluid between 39°C and 43°C. Argon gas is then circulated through the cryoprobes generating very low temperatures which freeze and destroy the affected tissue. Two cycles of freezing and thawing constitute the treatment.

The procedure takes between 1½ to 2 h. Patient is discharged on the same day or next morning with a urethral catheter for two to three weeks. The rates of complications such as impotence are higher and are highly operator-dependent.[10] Limited preliminary data are available about the efficacy of the third generation cryoablation system.

Zisman *et al.*,[8] reported three cases of urethral sloughing among 92 cryoablations performed. There were no fistulas and the overall complication rate was 8.7%. Among 36 patients with a nadir PSA level available, 31 (86%) had reached a nadir PSA lower than 0.5 ng/ml. The authors concluded that a longer follow-up was required to determine the efficacy of cryoablation.

Han *et al.*,[11] reported short-term (one year) results of 122 patients evaluated in a multi-institutional study. These patients were all treated strictly with the third generation cryoablation system. The PSA recurrence-free survival (PSA nadir <0.4 ng/ml) was 75% at one year. The rate of incontinence (patients who required pads daily) was 4.3%; an additional 5.1% had urge incontinence. There were no fistulas or strictures reported.

### Advantages:

1. Can be performed as day surgery under spinal anesthesia.
2. Can be repeated if there is local recurrence of cancer.

### Disadvantages:

1. Requires excellent surgical skills for consistently good results.
2. Has the highest propensity for impotence (85-95%); some studies show it to be as high as 76-100%.[14]
3. Requires a catheter for few weeks after treatment.
4. Sloughing of dead prostate tissue may require transurethral surgery for treatment in 5-10% of cases.
5. Short-term hormone blockade may be needed to shrink the prostate prior to cryotherapy in cases where the gland is too large.
6. The risk of incontinence even in the best hands may be as high as 5%.
7. Post operative pain syndrome occurs in a minority of patients (<5%).
8. Published data documenting long-term outcome is limited.

Further follow-up is necessary to assess whether third generation cryoablation systems are able to provide durable biochemical disease-free outcomes that are on par with conventional treatment modalities.

National Institute for Health and Clinical Excellence (NICE), UK has approved this technique in November 2005 for primary treatment of organ-confined prostate cancer as well as salvage therapy following failed radiotherapy.

## HIGH INTENSITY FOCUSED ULTRASOUND

The first reported application of HIFU was published in 1942[15] and this further evolved when William Fry *et al.* did experimental work on cat and monkey's brain by producing deep lesions in 1954-55.[16,17] During the 1950s and 1960s research into the use of HIFU in neurosurgery continued, but practical and technological limitations restricted their progress.[18,19] The initial work on its role in the treatment of the benign prostatic hypertrophy (BPH) began in the early 1990s and in 1996 Sanghavi *et al.*, did several safety and feasibility studies on canine prostate, utilizing HIFU and thermal mapping which led to treatment of BPH by this method and the ability to destroy the entire prostate tumor was reported by Madersbacher *et al.* in 1995.[20] Since then, various independent research is going on and this method is now approved for the treatment of prostatic cancer in Europe, China, Japan, Caribbean, Mexico and Latin America.

The quality which makes it desirable is the control and precision of HIFU. It allows the accomplished surgeon to accurately target the tissue to be destroyed without injuring adjacent tissue. High intensity focused ultrasound destroys tissue by heat, rather than by cavitation or mechanical shearing forces, and also this procedure utilizes transrectal ultrasound which is non-ionizing so tissues in the entry and exit path are not injured, but attenuation or weakening of the HIFU by the intervening tissues can occur. The density and content of the intervening tissues can affect the HIFU power. Bone or calcification can severely attenuate and even reflect the HIFU. Air not only attenuates HIFU, but interferes with imaging as well.[21]

### Indications

1. Patients with low clinical stage disease (cT1 to T2).
2. PSA less than 20 ng/ml.
3. Small prostate volumes (<40 ml) due to the limited focal length of HIFU.
4. Salvage therapy for patients in whom radiation therapy (either external radiation or brachytherapy) has failed. Brachytherapy seeds do not interfere with the energy transfer.
5. Local recurrences after radical prostatectomy.
6. Palliative therapy, debulking large tumors that are causing pain, bleeding and obstruction.

### Contraindications

1. Large gland size (>40 cc).
2. Extensive or very large calcifications in the gland.
3. Rectal stenosis interfering with the placement of probe.
4. History of rectal fistula.

### Procedure

High intensity focused ultrasound is performed as an outpatient procedure, usually under epidural anesthesia, with patient either in dorsal lithotomy position (Sonablate^®^ unit) or on his right side (Ablatherm^®^ unit). The HIFU probe is then placed into the rectum and multiple gland images are taken making sure that the top of the gland is included too. Then at the HIFU control panel, all of the images are reviewed and the treatment zones are defined and logged into the treatment computer. The entire prostate cannot be treated all at once, so the prostate is divided into treatment zones. Depending on the extent of the cancer, the side to side treatment zones may extend up to the edge or beyond the prostate capsule.

The probe emits a beam of ultrasound which is focused to reach a high intensity in the target area. Absorption of ultrasound energy creates an increase in temperature which causes coagulation of tissues within the focal area. A cooling balloon surrounding the probe protects the rectal mucosa from high temperature. An attractive advantage of HIFU is its low risk of morbidity, due to sudden, short bursts of the intensely focused ultrasound, which, along with the heat generated, are quickly absorbed by the target tissue, thereby protecting the surrounding tissues from damage.

The entire procedure takes around 90 min[42] depending on the gland size (average time 2-3 h).

After the procedure, urethral catheter is placed for one to two weeks.

For bigger gland size androgen blocking is done for three to six months for downsizing. Nowadays many patients undergo TURP immediately before HIFU under the same anesthesia.[42]

Table 1 presents the treatment side-effects of HIFU and compares them with three other leading treatments.[22,23]

**Table 1:** Data table comparing treatment side-effects[22–39]

	Rectal injury					
			
	Fistula %	Urgency %	Bleeding %	Diarrhea %	%	%
Radical prostatectomy	-	6-16	1-3	6-19	7-52	14-96
External beam radiation	-	19-43	13-17	12-42	0-15	50-61
Brachytherapy	0-3	-	4-11	-	0-19	14-66
Cryoablation	0-0.5	-	-	-	1-7	47-95
High intensity focused ultrasound (sonablate)	<0.5-5	-	-	-	0-2	28-30

Table 2, modified from Katz and Rewcastle, compares (1) the five-year biochemical disease-free survival rates as published since 1992 for five prostate cancer local treatments with (2) that published by Gelet *et al.*, for HIFU.[21,38–39]

**Table 2:** Five-year outcome data comparison (biochemical disease free survival)[21,39–40]

	Radical prostatectomy %	Cryotherapy %	Brachytherapy %	External beam radiation %	HIFU%
Low	76-98	60-92	78-89	81-86	70-71
Moderate	37-77	61-89	66-82	26-60	

Long term follow-up data is from papers by Chaussay, Thuroff[40] and Gelet *et al*.[39] It also includes the recent (2002) European data (including over 2000 patients) reported by Chaussay. In their overall case series, they have observed negative biopsy rates in 87.2% of patients and PSA value remained at their post HIFU nadir in 84.1% at one year.

In a study by Uchida[41] published in February 2005, a total of 132 consecutive patients with Stage T1c-2N0M0 localized prostate cancer underwent HIFU using Sonablate-500^®^. The five-year biochemical disease-free rate in all patients was 67%. The five-year biochemical disease-free rates for patients with a pretreatment PSA less than 10 ng/ml, 10.01 to 20.0 ng/ml, 20.01-30.0 ng/ml and more than 30.01 ng/ml were 88%, 67%, 34% and 13% (log rank test, *P* < 0.0001), respectively.

In another study by Uchida[42] published in Oct 2005, biochemical disease-free survival rates in patients with serum PSA of less than 10 ng/ml and 10-20 ng/ml were 75% and 78% (*P* = 0.6152). No viable tumor cells were noted in 68% of patients by postoperative prostate needle biopsy. Prostatic volume was decreased from 24.2 ml to 14.0 ml at six months after HIFU (*P* < 0.01).

### Complications

A detailed account of the complications encountered during three years of experience (315 treatments) is provided by Thuroff and Chaussay[43–45] and the most recent data describe stress incontinence in 13%, erectile dysfunction in 22% and urinary tract infection in only 5%.

## CONCLUSION

It is not possible to have a single gold standard therapy for all types of prostate cancer. Treatment options depend upon the age of patient, grade of tumor, expectations out of treatment and patient's choice governs the treatment actually to be given. It is the selection of a patient for a particular treatment option that decides how favorable the outcome is going to be. Both these techniques are relatively new and they look promising but both lack long-term data to prove their efficacy.

High intensity focused ultrasound seems to have the potential to become one of the therapies for the treatment of primary prostate cancer in the young. It is minimally invasive, takes a short time to complete the procedure and it can be done on an outpatient basis, therefore cuts cost. What makes it more promising is its ability to provide more accurate targeting of the sound beams sparing nerves. Studies done till now show comparable biochemical disease-free survival rates to established treatment options with much less side-effects. The technological advances will further refine the procedure as side-effects may represent actually a worst case scenario, as the series includes the first patients ever to undergo HIFU as a therapy for prostate cancer and many of them were treated with the original prototype HIFU. The use of 3D ultrasonography will further enhance the “vision” of the operating surgeon and a better nerve-sparing ablation can be performed. High intensity focused ultrasound definitely shows the potential to become the next generation treatment for primary prostate cancer but long-term studies are still needed to establish its safety and efficacy.
